# Esketamine vs. placebo combined with erector spinae plane block vs. intercostal nerve block on quality of recovery following thoracoscopic lung resection: a randomized controlled factorial trial

**DOI:** 10.1097/JS9.0000000000002060

**Published:** 2024-08-22

**Authors:** Jing-hui Hu, Zhang-zhen Zhong, Hai-jing Shi, Jie Wang, Shaomu Chen, Xi-sheng Shan, Hua-yue Liu, Hong Liu, Lingzhong Meng, Fu-hai Ji, Ke Peng

**Affiliations:** a Department of Anesthesiology, First Affiliated Hospital of Soochow University; b Institute of Anesthesiology, Soochow University; c Department of Anesthesiology, Suzhou Ninth People’s Hospital; d Department of Thoracic Surgery, First Affiliated Hospital of Soochow University, Suzhou, Jiangsu, People’s Republic of China; e Department of Anesthesiology and Pain Medicine, University of California Davis Health, Sacramento, California, USA; f Department of Anesthesia, Indiana University School of Medicine, Indianapolis, USA

**Keywords:** erector spinae plane block, esketamine, intercostal nerve block, multimodal analgesia, quality of recovery, thoracoscopic lung surgery

## Abstract

**Background:**

Multimodal analgesic strategy is pivotal for enhanced recovery after surgery. The objective of this trial was to assess the effect of subanesthetic esketamine vs. placebo combined with erector spinae plane block (ESPB) vs. intercostal nerve block (ICNB) on postoperative recovery following thoracoscopic lung resection.

**Materials and Methods:**

This randomized, controlled, 2×2 factorial trial was conducted at a university hospital in Suzhou, China. One hundred adult patients undergoing thoracoscopic lung surgery were randomized to one of four groups (esketamine-ESPB, esketamine-ICNB, placebo-ESPB, and placebo-ICNB) to receive i.v. esketamine 0.3 mg/kg or normal saline placebo combined with ESPB or ICNB using 0.375% ropivacaine 20 ml. All patients received flurbiprofen axetil and patient-controlled fentanyl. The primary outcome was quality of recovery (QoR) at 24 h postoperatively, assessed using the QoR-15 scale, with a minimal clinically important difference of 6.0.

**Results:**

The median age was 57 years and 52% were female. No significant interaction effect was found between esketamine and regional blocks on QoR (*P*=0.215). The QoR-15 score at 24 h was 111.5±5.8 in the esketamine group vs. 105.4±4.5 in the placebo group (difference=6.1, 95% CI: 4.0–8.1; *P*<0.001); 109.7±6.2 in the ESPB group vs. 107.2±5.6 in the ICNB group (difference=2.5, 95% CI: 0.2–4.9; *P*=0.033; not statistically significant after Bonferroni correction). Additionally, esketamine resulted in higher QoR-15 scores at 48 h (difference=4.6) and hospital discharge (difference=1.6), while ESPB led to a higher QoR-15 score at 48 h (difference=3.0).

**Conclusions:**

For patients undergoing thoracoscopic lung resection, subanesthetic esketamine improved QoR after surgery, while ICNB can be used interchangeably with ESPB as a component of multimodal analgesia.

## Introduction

Highlights

- This factorial trial assessed esketamine vs. placebo combined with ESPB vs. ICNB on recovery quality after thoracoscopic lung surgery.
- ICNB can be used interchangeably with ESPB as a component of multimodal analgesia during these surgical procedures.
- Esketamine improved our patients’ quality of recovery in a statistically and clinically significant manner.

Thoracoscopic surgery for lung resection is widely performed to facilitate enhanced recovery after surgery (ERAS)^1^. Compared with thoracotomy, thoracoscopic procedures are associated with lower morbidity, shorter hospital stay, and better quality of life^2,3^. Despite minimally invasive techniques, pain management after thoracoscopic surgery is still challenging. Inadequate pain control increases the risk of postoperative complications, impairs the quality of recovery (QoR), prolongs hospitalization, and may lead to long-term consequences such as chronic pain^4,5^. Thus, optimizing postoperative pain control is a priority in patients undergoing thoracoscopic lung surgery.

Opioids exert potent analgesic effects for patients undergoing surgery, but their use is not without side effects such as hyperalgesia, respiratory depression, nausea and vomiting, ileus, and cardiovascular events^6^. Opioid consumption reduction can mitigate the associated side effects and enhance recovery outcomes for surgical patients. Opioid-sparing anesthesia, regional analgesia, and multimodal pain management are essential elements of ERAS^7^. Esketamine is an N-methyl-D-aspartate antagonist that has been used as an adjunct to general anesthesia^8^. A subanesthetic dose of esketamine (typically 0.15–0.3 mg/kg) is clinically administered to improve postoperative pain management and decrease opioid consumption, while reducing its mental side effects^9,10^.

Since first described in 2016, the erector spinae plane block (ESPB) has been shown to reduce postoperative pain and improve recovery quality^11–13^. Owing to its safety and simplicity compared to epidural or paravertebral blocks, ESPB has been increasingly adopted in thoracic analgesia^14^. Intercostal nerve block (ICNB) is an effective, safe, and simple analgesic technique for thoracic surgery. There is evidence that ESPB provided superior analgesia and better clinical outcomes than ICNB^15^, whereas others argued that these two blocks were equally effective^16,17^.

In clinical practice, improving postoperative analgesia and decreasing opioid consumption can only be clinically meaningful when QoR is promoted following surgery. The 15-item QoR (QoR-15) scale is a widely used patient-centered global outcome measure of postoperative recovery^18,19^. Therefore, we designed this randomized factorial trial to compare esketamine with a normal saline placebo combined with ESPB or ICNB in patients undergoing thoracoscopic lung resection. We hypothesized that a subanesthetic dose of esketamine and the application of ESPB would contribute to enhanced postoperative QoR in these surgical patients.

## Methods

### Study design

This was a single-center, randomized, controlled, 2×2 factorial trial. Using a factorial design, we assessed the main effects of two interventions (esketamine and regional blocks) simultaneously and explored their possible interactions^20,21^. Patients were randomly treated with one of two study medications (esketamine or normal saline placebo) in combination with one of the two regional blocks (ESPB or ICNB). This resulted in four treatment combinations: esketamine-ESPB, esketamine-ICNB, placebo-ESPB, and placebo-ICNB.

### Ethical approval

This trial was approved by our institutional Ethics Committee (No. 2022-205) on 4 August 2022. The study protocol was registered in the Chinese Clinical Trial on 4 September 2022 before the first patient enrollment. All patients provided written informed consent on the day of the surgery or during the preoperative visit. This work has been reported in line with Consolidated Standards of Reporting Trials (CONSORT) Guidelines^22^.

### Inclusion and exclusion criteria

Inclusion criteria were age ≥18 years, ASA physical status I to III, and undergoing thoracoscopic lung surgery under general anesthesia. Exclusion criteria were BMI ≥35 kg/m^2^; emergency surgery; allergy to drugs used in this study; severe cardiopulmonary disease (myocardial infarction, heart failure, and respiratory failure); severe cerebrovascular disease (cerebral hemorrhage and stroke); severe liver or kidney disease (Child-Pugh grade C, renal replacement therapy); severe neurological disease (Parkinson’s disease, Alzheimer’s disease); use of antipsychotics, alcohol abuse, long-term use of opioids or other analgesics; uncontrolled hypertension; increased intracranial pressure, glaucoma, penetrating ocular trauma; or inability to understand the rating scales.

### Randomization and blinding

A researcher who did not participate in the subsequent study conducted the randomization in a ratio of 1:1:1:1 using the Sealed Envelope random sequence generator. The random list was then sealed in opaque envelopes. Immediately before surgery, a research nurse who was not involved in patient enrollment, perioperative care, or data collection opened the envelopes to assign patients to receive either esketamine or placebo as an adjunct analgesic and either ESPB or ICNB as a regional block. To achieve blinding of the esketamine groups, esketamine and placebo (0.9% normal saline) were distributed in identical 10 ml syringes. The regional block groups were not blinded. All patients and postoperative assessors were blinded to group allocation.

### Anesthesia

All patients underwent the standard ASA monitoring. General anesthesia was induced using i.v. sufentanil 0.3 µg/kg and propofol 1.5–2.0 mg/kg. Patients were administered i.v. rocuronium 0.6 mg/kg to facilitate double-lumen tube intubation for one-lung ventilation. Correct tube positioning was confirmed using fiberoptic bronchoscopy and auscultation. Anesthesia was maintained by inhalation of sevoflurane and titrated to bispectral index 40–60 (Medtronic). Intraoperative analgesia was achieved using additional doses of sufentanil 0.1 µg/kg, guided by the surgical pleth index within 20–50 (B650, GE Healthcare). The use of bispectral index and surgical pleth index to optimize anesthesia management has been reported in our recent study^23^.

Dexamethasone (5 mg) and ondansetron (4 mg) were i.v. administered to prevent postoperative nausea and vomiting (PONV). Other components of multimodal analgesia included i.v. flurbiprofen axetil (50 mg during surgery and every 12 h for the first two postoperative days) and 48 h patient-controlled i.v. fentanyl (10 µg/ml; 1 ml bolus and lockout time of 5 min). If patients still experienced pain with a visual analog scale (VAS, 0–10; 0 indicating no pain, 10 indicating the most severe pain) score ≥4, rescue analgesia was administered using additional i.v. fentanyl (50 µg). We applied a standardized institutional ERAS protocol for all patients undergoing thoracoscopic surgery, as described in the previous studies^23–25^.

### Study interventions

**Figure 1:**
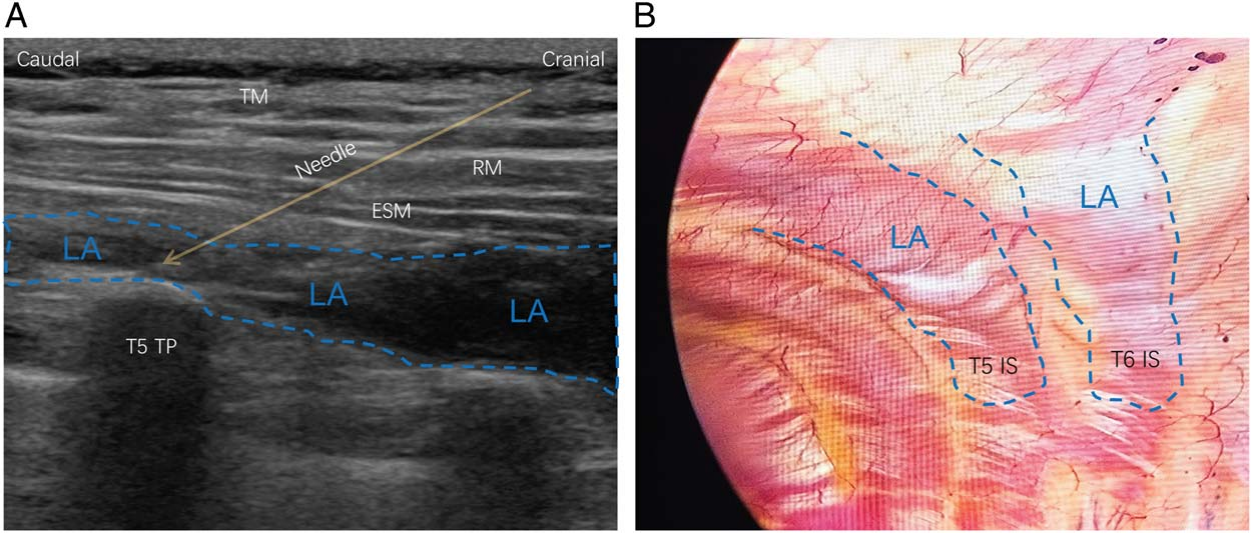
Erector spinae plane block and intercostal nerve block. (A) Ultrasound image of erector spinae plane block. (B) Thoracoscopic visualization of intercostal nerve block. ESM, erector spinae muscle; IS, intercostal space; LA, local anesthetic; RM, rhomboid muscle; TM, trapezius muscle.

After anesthesia induction and before surgical incision, a dose of 0.3 mg/kg esketamine i.v. was administered to patients in the esketamine groups, while an equivalent volume of normal saline was administered to the placebo groups. ESPB or ICNB with 0.375% ropivacaine 20 ml was performed in the lateral position at the end of the surgery. An aspiration test was performed to prevent inadvertent intravascular injections. ESPB was performed or supervised by an experienced attending anesthesiologist using the same ultrasound machine and linear transducer (SonoSite). Under ultrasound guidance, a 22-gauge block needle was advanced in-plane in a craniocaudal direction until it contacted the T5 transverse process, and we observed a linear spread of local anesthetic between the transverse processes and the erector spinae muscle (Fig. 1A). ICNB was performed by experienced surgeons under thoracoscopic visualization. Local anesthetic was injected 3–5 cm away from the spinous processes at the T3–T8 levels (3–4 ml for each intercostal space), and swelling was observed under thoracoscopy (Fig. 1B).

### Primary outcome

The primary outcome was QoR at 24 h postoperatively, assessed by blinded assessors using the Chinese version of the QoR-15 scale. QoR-15 is a widely used patient-centered global outcome measure of postoperative recovery^18,19,26^. The validity, reliability, and responsiveness of its Chinese version have been demonstrated^27^. To avoid potential observer bias, patients were left alone in the ward to complete the questionnaire in their own time. The average time to complete QoR-15 was ~2 min^27,28^. This scale comprises 15 questions on pain, physical comfort, physical independence, psychological state, and emotional state. The total score ranges from 0 to 150, with higher scores indicating better recovery. The updated minimal clinically important difference (MCID) for the QoR-15 score was 6.0^18,29^.

### Secondary outcomes

The secondary outcomes included QoR-15 scores at 48 h and at hospital discharge; VAS pain scores at rest and on coughing in the post-anesthesia care unit (PACU) and at 24 h and 48 h; patient-controlled fentanyl consumption during 0–48 h; need for rescue analgesia during 0–48 h; hypotension, hypertension, bradycardia, tachycardia; PONV; adverse effects (headache, dizziness, nightmare, sleep disorder, and mood disorder); severe complications (myocardial infarction, heart failure, respiratory failure, stroke, gastrointestinal bleeding, sepsis, reoperation, and death); and length of postoperative hospital stay.

### Sample size

In our preliminary observation, the mean±SD QoR-15 score at 24 h after thoracoscopic lung surgery was 110.2±6.4 in patients who received esketamine vs. 106.5±4.8 in those who received placebo (*n*=10 in each group), and 113.5±7.5 in patients receiving ESPB vs. 107.9±7.0 in those receiving ICNB (*n*=8 in each group). These results are in line with those of a recent study in which the mean QoR-15 scores at 24 h ranged from 102 to 114^28^. Based on these pilot data, we estimated the sample size of this factorial trial using the PASS software (version 11.0.7; NCSS, LCC). To determine the main effect of each intervention at a significance level of 0.025 and power of 80%, 92 patients were required to assess esketamine vs. placebo (*n*=46 in each group with either regional block) and 68 patients for ESPB vs. ICNB (*n*=34 in each group with either esketamine or placebo). After accounting for a possible dropout rate of 5%, we set the sample size to 100, with 25 participants in each group.

### Statistical analysis

The Shapiro–Wilk test was used to assess normality. Data are presented as mean±SD, median (interquartile range [IQR]), or number (%), depending on type and distribution. Given the factorial design, two-way analysis of variance (ANOVA) with QoR-15 score at 24 h as the outcome variable was used to assess whether an interaction existed between the two interventions. If there was no significant interaction, treatment effect estimates were summarized comparing esketamine vs. placebo and ESPB vs. ICNB. If the interaction was significant, the effect of each intervention should be assessed within the levels of the other interventions.

To assess the main effects, data were analyzed using unpaired *t*-test, Mann–Whitney rank-sum test, *χ* ^2^ test, or Fisher’s exact test, as appropriate. Between-group differences were further presented as the mean or median difference and 95% CI. With an overall significance level of 0.05, for the primary outcome in the two comparisons, the significance criterion was 0.025 for each interventional effect (0.05/2, Bonferroni correction). We did not perform multiple comparison corrections for secondary outcomes. Prespecified exploratory analyses were conducted across four factorial groups (representing each of the esketamine and regional analgesia combinations) using one-way ANOVA, Kruskal–Wallis test, or *χ* ^2^ test, as appropriate. Primary analyses were conducted for all randomized patients with available outcome data. There was no plan for the imputation of missing data. Data were analyzed using the GraphPad Prism software (version 9.0.0; GraphPad).

## Results

### Study flow and patient characteristics

**Figure 2:**
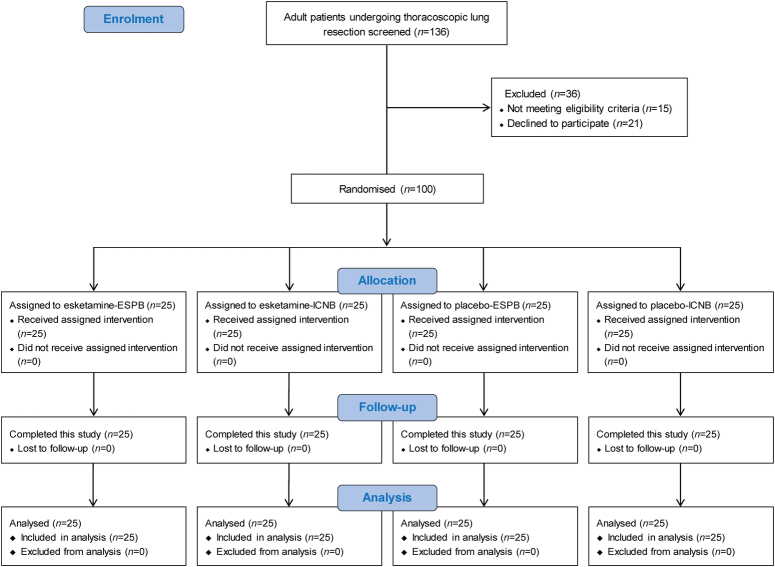
CONSORT flow diagram of patients in this 2×2 factorial trial.

A total of 136 patients were screened between September and December 2022 (Fig. 2). Of these, 100 were randomized: 50 were assigned to esketamine, of whom 25 were assigned to ESPB and 25 to ICNB; 50 were assigned to placebo, of whom 25 were assigned to ESPB and 25 to ICNB. All patients completed the scheduled study intervention and assessment without missing data.

Overall, the median (IQR) age was 57 (48–62) years, and 52% of the patients were female (Table 1). Baseline characteristics (demographics, ASA physical status, comorbidities, and pulmonary function) and surgical data (intraoperative sufentanil dose, type of surgery, and surgical time) were balanced among the groups.

**Table 1:** Baseline characteristics and surgical data.

	Esketamine-ESPB (*n*=25)	Esketamine-ICNB (*n*=25)	Placebo-ESPB (*n*=25)	Placebo-ICNB (*n*=25)	*P*
Age (years)	54 (49.5–59.5)	58 (47–60.5)	54 (46.5–63.5)	58 (46–62.5)	0.994
Sex					
Female	13 (52)	16 (64)	11 (44)	12 (48)	0.523
Male	12 (48)	9 (36)	14 (56)	13 (52)	0.523
Weight (kg)	63±9.6	64.4±10.5	64.5±10.5	66.1±10.2	0.764
Height (cm)	163±7.3	164±7.2	164±7.4	166±7.7	0.611
BMI (kg/m^2^)	23.5±2.3	23.8±2.6	23.8±2.7	23.9±1.9	0.966
Current smoker	6 (24)	3 (12)	5 (20)	6 (24)	0.682
ASA physical status					
1	14 (56)	15 (60)	9 (36)	12 (48)	0.339
2	10 (40)	8 (32)	14 (56)	13 (52)	0.299
3	1 (4)	2 (8)	2 (8)	0	0.538
Comorbidities					
Hypertension	9 (36)	7 (28)	6 (24)	7 (28)	0.820
Diabetes	3 (12)	4 (16)	3 (12)	5 (20)	0.531
COPD	2 (8)	0	1 (4)	2 (8)	0.837
Cardiovascular disease	3 (12)	1 (4)	4 (16)	3 (12)	0.668
Anemia	2 (8)	2 (8)	1 (4)	0	0.151
Preoperative pulmonary function					
Predicted percentages of FVC (%)	105±15	104±11.5	107±9.8	99.1±11.7	0.150
Predicted percentages of FEV_1_ (%)	101±17.7	101±11.3	102±10.	95.9±15.6	0.444
FEV_1_ to FVC ratio (%)	98.6±10.4	100±6.0	98.2±6.6	97.0±12.8	0.723
Predicted percentages of MVV (%)	96.7±23.1	97.5±17.2	101±21.6	91.7±17.7	0.396
Intraoperative sufentanil (μg)	40 (40–50)	45 (40–50)	50 (40–50)	50 (40–50)	0.140
Surgical type					
Wedge resection	12 (48)	11 (44)	13 (52)	15 (60)	0.705
Segmentectomy	9 (36)	8 (32)	8 (32)	7 (28)	0.947
Lobectomy	4 (16)	6 (24)	4 (16)	3 (12)	0.718
Surgical time (min)	124±39	118±35	143±53	134±49	0.219

Data are mean±SD, median (IQR), or n (%).

COPD, chronic obstructive pulmonary disease; ESPB, erector spinae plane block; FEV_1_, forced expiratory volume in 1 s; FVC, forced vital capacity; ICNB, intercostal nerve block; MVV, maximal voluntary ventilation.

### Primary outcome

By using the factorial design, no significant interaction was found between the esketamine and regional block groups (*P*=0.215). Therefore, the results of the two main effects were analyzed and presented separately (Table 2).

**Table 2:** Primary and secondary outcomes.

	Use of esketamine				Use of regional block			
	Esketamine (*n*=50)	Placebo (*n*=50)	Difference or RR (95% CI)	*P*	ESPB (*n*=50)	ICNB (*n*=50)	Difference or RR (95% CI)	*P*
Primary outcome								
QoR-15 score at 24 h	111.5±5.8	105.4±4.5	6.1 (4.0–8.1)	<0.001	109.7±6.2	107.2±5.6	2.5 (0.2–4.9)	0.033
Secondary outcomes								
QoR-15 score at 48 h	120±5.5	115.4±3.9	4.6 (2.7–6.5)	<0.001	119.2±5.2	116.2±5.0	3.0 (0.9–5.0)	0.004
QoR-15 score at hospital discharge	135.6±4.3	133.9±3.9	1.6 (0–3.3)	0.049	135.1±4.2	134.3±4.2	0.8 (−0.9–2.5)	0.342
VAS pain scores at rest								
In PACU	2 (2–3)	2 (2–3)	0 (−1–0)	0.163	2 (2–3)	2 (2–3)	0 (0–0)	0.469
At 24 h	2 (2–3)	3 (2–4)	−1 (−1–0)	0.207	2.5 (2–3)	3 (2–3.25)	−0.5 (−1–0)	0.568
At 48 h	2.5 (2–3)	3 (2–3)	−0.5 (−1–0)	0.135	2 (2–3)	2 (2–3)	0 (0–0)	0.471
VAS pain scores on coughing								
In PACU	3 (3–4)	4 (3–4)	−1 (−1–0)	0.009	3 (3–4)	3 (3–4)	0 (0–0)	0.886
At 24 h	4 (3–5)	4 (4–5)	0 (−1–0)	0.013	4 (3–5)	4 (3.75–5)	0 (−1–0)	0.142
At 48 h	4 (3–4)	4 (3–4)	0 (−1–0)	0.084	4 (3–4)	4 (3–4)	0 (−1–0)	0.191
Fentanyl consumption 0–48 h (μg)	560 (420–900)	635 (500–860)	−75 (−140–40)	0.365	600 (428–900)	615 (500–900)	−15 (−120–60)	0.497
Rescue analgesia 0–48 h	7 (14)	13 (26)	0.54 (0.24–1.20)	0.134	9 (18)	11 (22)	0.82 (0.38–1.77)	0.617
Hypotension	7 (14)	12 (24)	0.58 (0.25–1.32)	0.203	9 (18)	10 (20)	0.90 (0.41–1.99)	0.799
Hypertension	5 (10)	6 (12)	0.83 (0.29–2.42)	0.749	7 (14)	4 (8)	1.75 (0.58–5.34)	0.525
Bradycardia	7 (14)	11 (22)	0.64 (0.27–1.46)	0.298	7 (14)	10 (20)	0.70 (0.29–1.64)	0.425
Tachycardia	3 (6)	5 (10)	0.60 (0.16–2.16)	0.715	5 (10)	3 (6)	1.67 (0.46–6.07)	0.715
PONV	14 (28)	17 (34)	0.82 (0.46–1.47)	0.517	18 (36)	13 (26)	1.39 (0.77–2.52)	0.280
Adverse effects	9 (18)	14 (28)	0.64 (0.31–1.32)	0.235	10 (20)	13 (26)	0.77 (0.38–1.56)	0.476
Severe complications	0	0	–	–	0	0	–	–
Length of postoperative hospital stay (days)	4 (3–5)	4 (2.75–5)	0 (−1–1)	0.835	4 (3–5)	3 (3–4)	1 (0–1)	0.062

Data are mean±SD, median (IQR), or n (%).

Adverse effects included headache, dizziness, nightmare, sleep disorder, and mood disorder.

Severe complications included myocardial infarction, heart failure, respiratory failure, stroke, gastrointestinal bleeding, sepsis, reoperation, and death.

ESPB, erector spinae plane block; ICNB, intercostal nerve block; PACU, postanesthesia care unit; PONV, postoperative nausea and vomiting; QoR, quality of recovery; RR, relative risk; VAS, visual analog scale.

**Figure 3:**
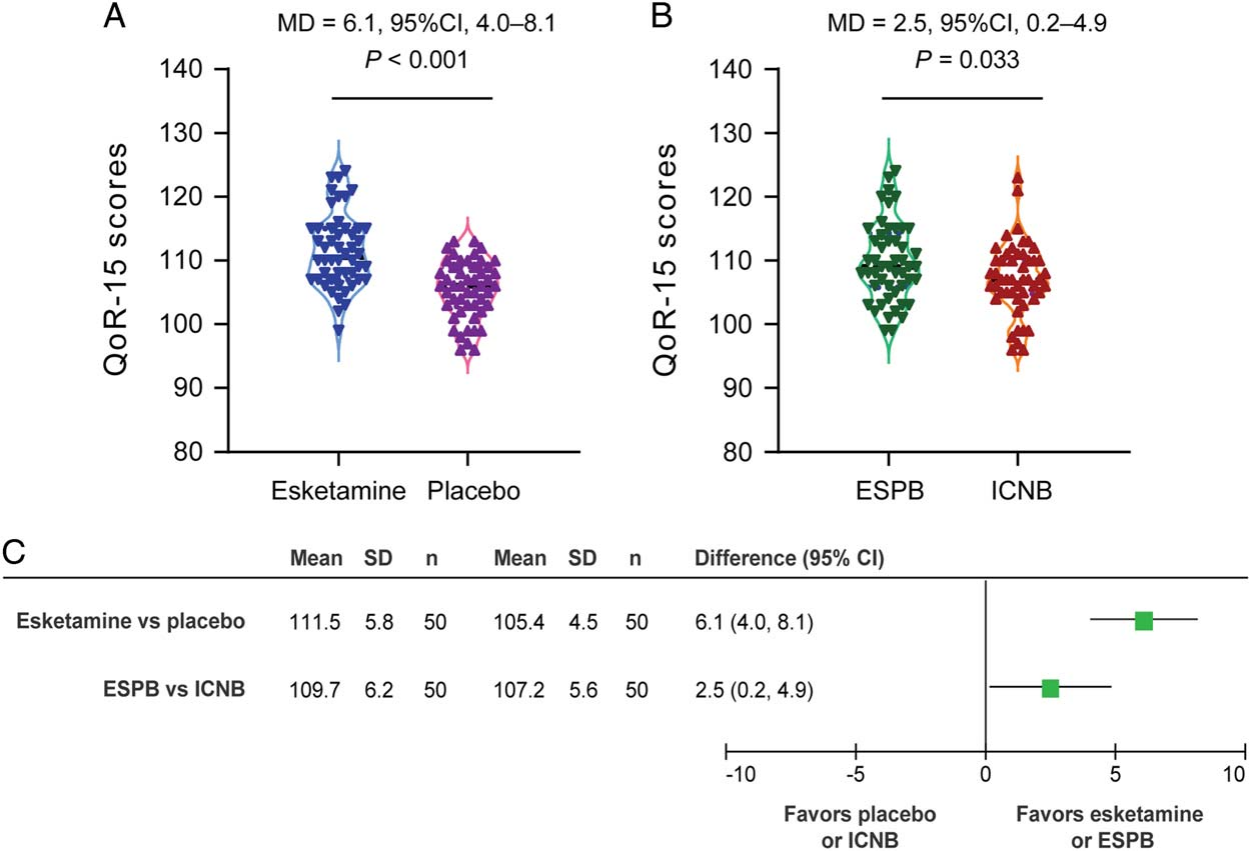
QoR-15 scores at 24 h postoperatively. (A) Esketamine vs placebo. (B) ESPB vs ICNB. (C) Effect size of esketamine vs placebo and ESPB vs ICNB. ESPB, erector spinae plane block; ICNB, intercostal nerve block; QoR-15, Quality of Recovery-15.

The QoR-15 score at 24 h postoperatively was 111.5±5.8 in patients who received esketamine, which was higher than 105.4±4.5 in patients who received placebo (difference=6.1, 95% CI: 4.0–8.1; *P*<0.001; Fig. 3A). The QoR-15 score at 24 h was 109.7±6.2 in patients with ESPB vs. 107.2±5.6 in those with ICNB (difference=2.5, 95% CI: 0.2–4.9; *P*=0.033, not statistically significant; Fig. 3B). For esketamine vs. placebo, the between-group difference of the treatment effect crossed the MCID of 6.0; for ESPB vs. ICNB, the 95% CI upper limit did not reach the MCID (Fig. 3C).

### Secondary outcomes

The use of esketamine led to higher QoR-15 scores at 48 h (difference=4.6, 95% CI: 2.7–6.5) and at hospital discharge (difference=1.6, 95% CI: 0–3.3), while ESPB was associated with a higher QoR-15 score at 48 h (difference=3.0, 95% CI: 0.9–5.0); however, all these differences were not clinically significant (Table 2).

VAS pain scores at rest were comparable between the esketamine and placebo groups as well as between the ESPB and ICNB groups. Esketamine was associated with a lower VAS pain score on coughing in the PACU (difference=−1, 95% CI: −1–0). Postoperative fentanyl consumption, rescue analgesia, hemodynamic events with interventions, PONV, adverse effects, and length of postoperative hospital stay were similar between the groups. None of the patients developed any severe complications during the study.

### Exploratory analysis

In the prespecified exploratory analysis of outcomes across all four groups, there were significant differences in QoR-15 scores at 24 and 48 h postoperatively (*P*<0.001) (Table 3). Using esketamine-ESPB as the reference category, there were statistically significant differences in QoR-15 scores at 24 h compared to esketamine-ICNB (difference=3.8, 95% CI: 0.4–7.2), placebo-ESPB (difference=7.3, 95% CI: 3.9–10.7), and placebo-ICNB (difference=8.6, 95% CI: 5.2–12.0). At 48 h postoperatively, esketamine-ESPB was also associated with higher QoR-15 scores than esketamine-ICNB (difference=3.8, 95% CI: 0.8–6.9), placebo-ESPB (difference=5.4, 95% CI: 2.4–8.5), and placebo-ICNB (difference=7.6, 95% CI: 4.5–10.6).

**Table 3:** Exploratory analyses of outcomes across four groups.

	Esketamine-ESPB (*n*=25)	Esketamine-ICNB (*n*=25)	Placebo-ESPB (*n*=25)	Placebo-ICNB (*n*=25)	*P*
QoR-15 score at 24 h	113.4±5.7	109.6±5.3^a^	106.1±4.2^b^	104.8±4.8^c^	<0.001
QoR-15 score at 48 h	121.9±5.4	118.1±4.9^d^	116.4±3.1^e^	114.4±4.3^f^	<0.001
QoR-15 score at hospital discharge	136.6±3.5	134.5±4.9	133.7±4.4	134.2±3.4	0.067
VAS pain scores at rest					
In PACU	2 (2–3)	2 (2–3)	2 (2–3)	2 (2–3)	0.437
At 24 h	2 (2–3)	2 (2–3)	3 (2–3.5)	3 (2–4)	0.528
At 48 h	2 (2–3)	3 (2–3)	3 (2–3)	3 (2–3)	0.415
VAS pain scores on coughing					
In PACU	3 (3–3.5)	3 (3–4)	4 (3–5)	4 (3–4)	0.067
At 24 h	4 (3–4.5)	4 (3–5)	4 (4–5)	5 (4–6)	0.041
At 48 h	3 (3–4)	4 (3–4)	4 (3–4)	4 (3–4.5)	0.159
Fentanyl consumption 0–48 h (μg)	480 (330–900)	560 (500–900)	640 (540–870)	630 (490–860)	0.549
Rescue analgesia 0–48 h	3 (12)	4 (16)	6 (24)	7 (28)	0.475
Hypotension	4 (16)	3 (12)	5 (20)	7 (28)	0.518
Hypertension	3 (12)	2 (8)	4 (16)	2 (8)	0.771
Bradycardia	3 (12)	4 (16)	4 (16)	6 (24)	0.718
Tachycardia	2 (8)	1 (4)	3 (12)	2 (8)	0.780
PONV	8 (32)	6 (24)	10 (40)	7 (28)	0.651
Adverse effects	4 (16)	5 (20)	6 (24)	8 (32)	0.577
Severe complications	0	0	0	0	–
Length of postoperative hospital stay (days)	3 (2–5.5)	4 (3–5)	4 (3.5–5)	3 (3–4)	0.094

Data are mean±SD, median (IQR), or n (%).

Adverse effects included headache, dizziness, nightmares, sleep disorder, and mood disorder.

Severe complications included myocardial infarction, heart failure, respiratory failure, stroke, gastrointestinal bleeding, sepsis, reoperation, and death.

^a^ Esketamine-ESPB vs esketamine-ICNB: difference=3.8, 95% CI: 0.4–7.2, *P*=0.025.

^b^ Esketamine-ESPB vs placebo-ESPB: difference=7.3, 95% CI: 3.9–10.7, *P*<0.001.

^c^ Esketamine-ESPB vs placebo-ICNB: difference=8.6, 95% CI: 5.2–12.0, *P*<0.001.

^d^ Esketamine-ESPB vs esketamine-ICNB: difference=3.8, 95% CI: 0.8–6.9, *P*=0.010.

^e^ Esketamine-ESPB vs placebo-ESPB: difference=5.4, 95% CI: 2.4–8.5, *P*<0.001.

^f^ Esketamine-ESPB vs placebo-ICNB: difference=7.6, 95% CI: 4.5–10.6, *P*<0.001.

ESPB, erector spinae plane block; ICNB, intercostal nerve block; PACU, postanesthesia care unit; PONV, postoperative nausea and vomiting; QoR, quality of recovery; VAS, visual analog scale.

## Discussion

In this 2×2 randomized factorial trial, there was no significant interaction between esketamine and the regional blocks; thus, their treatment effects were assessed separately. For the primary outcome measured by QoR-15 at 24 h postoperatively, subanesthetic esketamine vs. placebo led to a statistically higher QoR-15 score, with a between-group difference of 6.1; the effect of ESPB vs. ICNB on the 24 h QoR-15 score was not statistically significant, with a between-group difference of 2.5. Based on these findings and considering an MCID of 6.0, subanesthetic esketamine led to a statistically and clinically significant improvement of QoR in patients undergoing thoracoscopic lung resection, while ESPB compared with ICNB did not have a significant impact on QoR in these patients.

Patients value rapid recovery after surgery, a comfortable experience during the perioperative period, and early return to normal activities. QoR measurements, including QoR-9, QoR-40, and QoR-15, are widely used for global assessment of patients’ postoperative recovery^26,30,31^. The QoR-40 and QoR-15 scales have been validated across various surgical populations with translation into 20 languages including Chinese^27,31^. Compared with QoR-40, the QoR-15 scale is easier to use and quicker to complete^26^. According to the COSMIN guidelines, a systematic review demonstrated that the QoR-15 scale has good validity, internal consistency, and unidimensionality as a measuring instrument for postoperative QoR^32^. When used in Chinese patients, the QoR-15 Chinese version is also reliable, responsive, and easy to use with satisfactory psychometric properties^27^. The MCID represents the smallest change in score, indicating a clinically meaningful change in the health state. Dr. Myles and colleagues recommended a threshold of 8.0 for the QoR-15 scale^18^. In 2021, they updated the MCID of QoR-15 to a value of 6.0 after undertaking further analysis^29^. Therefore, we chose an MCID of 6.0 for the QoR-15 score in this trial.

Esketamine is an S-enantiomer of racemic ketamine with high analgesic potency^33^. Subanesthetic esketamine has been used in general anesthesia to potentiate analgesia and avoid side effects^34^. In a previous study, esketamine (0.25 mg/kg after induction and 0.125 mg/kg/h intraoperatively) compared with placebo led to a mean increase in the 48-h QoR-40 score of 7, lower pain scores, and a lower requirement for rescue analgesics after thoracoscopic surgery^35^. Yuan *et al*.^36^ also suggested that intraoperative esketamine infusion of 0.25 mg/kg/h decreased postoperative hydromorphone use and improved QoR after thoracic surgery. A recent trial showed that a single subanesthetic dose of esketamine (0.3 mg/kg) after anesthesia induction reduced postoperative pain and anxiety in patients undergoing breast and thyroid surgery^37^, whereas Zhou *et al*.^38^ found that esketamine (0.5 mg/kg) did not significantly reduce acute and chronic pain or analgesic consumption following thoracoscopic surgery. In this study, we observed a statistically significant reduction in QoR-15 scores by esketamine treatment (difference=6.1, 95% CI: 4.0–8.1). Considering the MCID of 6.0, this improvement in QoR was also clinically meaningful.

Regional analgesia is an essential component of postoperative multimodal pain management and has been used to enhance QoR in patients undergoing video-assisted thoracic surgery^39^. These regional analgesic techniques include epidural block, paravertebral block, serratus anterior plane block, ESPB, and ICNB^40^. According to the PROSPECT guidelines, ESPB or thoracic paravertebral block is recommended as a first-choice option in thoracoscopic surgery^40,41^. The use of ESPB can provide adequate analgesic effects through the injection of local anesthetics in the erector spinae fascial plane acting on both the ventral and dorsal rami of the spinal nerves, and its efficacy in reducing postoperative pain and improving QoR has been well defined^12,42^. ICNB is a traditional analgesic approach used in thoracic surgeries. A recent meta-analysis demonstrated that single-injection ICNB was clinically noninferior to epidural or paravertebral block during the first 24 h after thoracic surgery^43^. Compared with ESPB, ICNB has been shown to have equal effects on 24 h postoperative pain and morphine consumption after thoracoscopic surgery^16^. Our results are in line with those of previous studies, showing that ESPB and ICNB had comparable effects on QoR after thoracoscopic lung resection surgery.

To the best of our knowledge, this is the first factorial trial to assess the effects of two anesthetic treatments (esketamine and regional blocks) on the QoR in patients who underwent thoracoscopic surgery. This study has several limitations. First, our patients had a median age of 57 years, and further studies to explore the effects of treatment on QoR in elderly patients are warranted. Second, there was no blinding of the regional block groups; however, postoperative assessors were blinded to group allocation. Third, although successful blocks were achieved under ultrasound guidance or thoracoscopic visualization, we did not measure the extent of the blocks because our patients were under general anesthesia. Finally, as this was a single-center study, the generalizability of our findings should be tested in future studies.

## Conclusion

Subanesthetic esketamine led to an improvement in QoR after thoracoscopic lung surgery in a statistically and clinically significant manner, while ESPB and ICNB were equally effective as a component of multimodal analgesia.

## Ethical approval

This trial was approved by the Ethics Committee of the First Affiliated Hospital of Soochow University, China (No. 2022-205; dated 4 August 2022).

## Consent

All patients provided written informed consent on the day of the surgery or during the preoperative visit.

## Source of funding

This study was supported by the Suzhou Medical Health Science and Technology Innovation Project (SKY2022136), Key Medical Research Projects in Jiangsu Province (ZD2022021), and Six Talent Peaks Project in Jiangsu Province (WSN-022). The funders had no role in the study design, data collection, data analysis, interpretation, or writing of this manuscript.

## Author contribution

J.H.H., Z.Z.Z., F.H.J., and K.P.: study design; J.H.H., Z.Z.Z., H.J.S., X.S.S., and H.Y.L: data acquisition; J.H.H., Z.Z.Z., H.J.S., and J.W.: manuscript drafting; J.H.H., Z.Z.Z., and K.P.: statistical analysis; X.S.S., H.Y.L., H.L., L.M., F.H.J., and K.P.: manuscript revision. All authors contributed in final approval of the version to be submitted and data interpretation.

## Conflicts of interest disclosure

The authors declare no conflicts of interest.

## Research registration unique identifying number (UIN)

The study protocol was registered in the Chinese Clinical Trial Registry (No. ChiCTR2200063334; dated September 4, 2022; https://www.chictr.org.cn/showprojEN.html?proj=177687) before the first patient enrollment.

## Guarantor

Ke Peng, MD, PhD, Vice Chair for Research and Associate Professor, Department of Anesthesiology, First Affiliated Hospital of Soochow University, 188 Shizi Street, Suzhou, Jiangsu 215006, People’s Republic of China. Tel.: +86 512 67780055. E-mail: pengke0422@163.com.

Fu-hai Ji, MD, PhD, Chair and Professor, Department of Anesthesiology, First Affiliated Hospital of Soochow University, 188 Shizi Street, Suzhou, Jiangsu 215006, People’s Republic of China. Tel.: +86 512 67780056. E-mail: jifuhaisuda@163.com.

## Data availability statement

Data can be obtained upon scientifically sound request from the corresponding author at pengke0422@163.com or jifuhaisuda@163.com.

## Provenance and peer review

Not applicable.
